# House Ventilation and Warming

**Published:** 1890-10-11

**Authors:** 


### House Ventilation and Warming.

The object of this book* is to urge that when a dwelling-house of a certain size and pretension is to be constructed, special arrangements should be introduced to secure at all times comfortable warmth and sufficient ventilation in all the living rooms, and that this can only be effected by a general plan which shall include a central warmed hall from which all the rooms are to received their air supply, and a single foul-air chamber or outlet flue, communicating with all the rooms by opening in the ceiling, and especially in the cornice, and the movement of the air in which is to be produced or acclerated by utilizing the waste heat of the kitchen chimney.

The methods proposed for carrying out this plan are more especially adapted to the climatic conditions of England, and to the habits and tastes of the middle-class. The warming of the fresh air introduced is not relied upon for heating the house as is so often done in America, but direct radiation from open fire-places or grates is to be provided in addition for each living-room. The warming of the fresh air introduced is recommended because unless it is so warmed there is a constant liability to the production of cold and unpleasant, if not dangerous, currents and draughts, not only from whatever inlets may be specially provided for fresh air, but also from cracks and crevices around doors and windows, and the authors therefore recommend that all the incoming air shall be warmed to about 65 degrees by hot water pipes, while the loss of heat due to radiation from windows and outer walls shall be made good by open grates.

No attention is paid to the fact that in the great majority of such dwelling houses as are specially considered in this book, the amount of air which passes directly through the walls and ceilings is very large, so great in fact that if the walls are not made nearly or entirely air-tight by paint or paper the amount of air which they transmit is nearly sufficient to secure satisfactory ventilation.

The principle upon which the amount of air required is calculated in this work is quite erroneous, although, curiously enough, the gross result obtained is nearly correct. The error consists in allowing only 15 cubic feet per minute as the allowance for each person, whereas 45 cubic feet per minute are required to so dilute the exhalations that the air in the room shall give no perceptible odour to a person entering it from the outer air. On the other hand, it is not necessary to make a totally separate and distinct allowance of air supply for each fire-place and gas burner in inhabited rooms, for the very simple reason that the air which has been so vitiated by the respiration of human beings that it should not be rebreathed before purification, may, nevertheless, be a very good source of supply of oxygen to burning fuel or gas.

The conclusion for the particular house described in the book, viz., that the fresh air inlet should be capable of replenishing the whole house three times every hour, is about correct; but it is made to depend more on the number of fire-places and gas-burners than upon the number of persons to be supplied.

In their preface to this edition the authors remark that the main general principle proposed by them in the first edition in 1872 is still far from being generally admitted or put into practice. This is undoubtedly true, in fact we doubt whether there are fifty houses in existence which have the central hall—the inlets and outlets both at the ceiling, and the elaborate system of aspirating tubes connected with the kitchen chimney—which combination is the only new principle of their original plan. There are many ways of heating and ventilating a dwelling-house besides the one urged in this volume.

Nevertheless we believe that the book has been a useful one to architects, if not to housebuilders, and that this new edition will be found to be suggestive in many ways even though the details may not be fully approved. The appendix, giving the results of some observations and experiments made upon temperatures and air currents in a house built upon this plan, contains some data which wil be of interest to the architect and heating engineer.

*Health and Comfort in House-building; or Ventilation with Warm Air by Self-acting Suction Power, by J. Drysdale, M.D., and J. W. Hayward, M.D. Third edition. (London: E. and F. N. Spon, 1890.)

